# Heterologous overexpression of *Glomerella cingulata *FAD-dependent glucose dehydrogenase in *Escherichia coli *and *Pichia pastoris*

**DOI:** 10.1186/1475-2859-10-106

**Published:** 2011-12-12

**Authors:** Christoph Sygmund, Petra Staudigl, Miriam Klausberger, Nikos Pinotsis, Kristina Djinović-Carugo, Lo Gorton, Dietmar Haltrich, Roland Ludwig

**Affiliations:** 1 Food Biotechnology Laboratory, Department of Food Sciences and Technology, BOKU-University of Natural Resources and Life Sciences, Muthgasse 18/2 Wien, Austria; 2 Department of Structural and Computational Biology, Max F. Perutz Laboratories, University of Vienna, Campus Vienna Biocenter 5, A-1030 Vienna, Austria; 3 Department of Biochemistry, Faculty of Chemistry and Chemical Technology, University of Ljubljana, Aškerčeva 5, 1000 Ljubljana, Slovenia; 4 Department of Biochemistry and Structural Biology, Lund University, P. O. Box 124, 22100 Lund, Sweden

## Abstract

**Background:**

FAD dependent glucose dehydrogenase (GDH) currently raises enormous interest in the field of glucose biosensors. Due to its superior properties such as high turnover rate, substrate specificity and oxygen independence, GDH makes its way into glucose biosensing. The recently discovered GDH from the ascomycete *Glomerella cingulata *is a novel candidate for such an electrochemical application, but also of interest to study the plant-pathogen interaction of a family of wide-spread, crop destroying fungi. Heterologous expression is a necessity to facilitate the production of GDH for biotechnological applications and to study its physiological role in the outbreak of anthracnose caused by *Glomerella *(*anamorph Colletotrichum) spp*.

**Results:**

Heterologous expression of active *G. cingulata *GDH has been achieved in both *Escherichia coli *and *Pichia pastoris*, however, the expressed volumetric activity was about 4800-fold higher in *P. pastoris*. Expression in *E. coli *resulted mainly in the formation of inclusion bodies and only after co-expression with molecular chaperones enzymatic activity was detected. The fed-batch cultivation of a *P. pastoris *transformant resulted in an expression of 48,000 U L^-1 ^of GDH activity (57 mg L^-1^). Recombinant GDH was purified by a two-step purification procedure with a yield of 71%. Comparative characterization of molecular and catalytic properties shows identical features for the GDH expressed in *P. pastoris *and the wild-type enzyme from its natural fungal source.

**Conclusions:**

The heterologous expression of active GDH was greatly favoured in the eukaryotic host. The efficient expression in *P. pastoris *facilitates the production of genetically engineered GDH variants for electrochemical-, physiological- and structural studies.

## Background

FAD-dependent glucose dehydrogenase (GDH, EC 1.1.99.10, D-glucose:acceptor 1-oxidoreductase) was first discovered in 1951 in *Aspergillus oryzae *[1] but remained a relatively little investigated enzyme. In the following decades, only a few FAD-dependent GDHs were characterized from the bacterium *Burkholderia cepacia *[2], the larvae of the moth *Manduca sexta *(tobacco hornworm) [3] and the fly *Drosophila melanogaster *[4]. Since the application of FAD-dependent GDH as electrode catalyst in glucose biosensors [2] and for biofuel cell anodes [5] was published and promoted, more attention was drawn to this enzyme, and new members were identified and characterized, e.g. from the fungi *A. terreus *[6], *A. oryzae *[1,7,8] and *Penicillium lilacinoechinulatum *[9]. The advantages of FAD-dependent GDH for their use in glucose biosensors are high turnover rates and a good stability. Moreover, its oxidative half-reaction is unaffected by oxygen, whereas the oxygen turnover in glucose oxidase-based electrodes reduces the electron yield and produces hydrogen peroxide which degrades the biocatalyst. In comparison with pyrroloquinoline quinone (PQQ)-dependent GDHs a lower redox potential of FAD-dependent GDH is noteworthy. Two big producers of glucose biosensors, Abbott and Bayer, already implemented FAD-dependent GDHs in some of their products. A novel member of the small family of FAD-dependent GDHs was recently discovered in the plant pathogenic fungus *Glomerella cingulata *(anamorph *Colletotrichum gloeosporoides*) and characterized [10]. It is an extracellular, glycosylated enzyme showing a narrow substrate specificity with β-D-glucose and D-xylose as substrates, which are oxidized at the anomeric carbon atom. The electrons are transferred to quinones, phenoxy radicals, redox dyes and iron complexes such as ferricyanide and ferrocenium hexafluorophosphate, but not to molecular oxygen. The biological function of this GDH is still unclear but a role during fungal attack on the host-plant is proposed. By reducing quinones and phenoxy radicals GDH is able to neutralize the action of plant laccases, phenoloxidases or peroxidases, which are used by infected plant tissues to parry the fungal attack.

Despite the enormous biotechnological relevance of FAD-dependent GDHs there are only scarce reports about their heterologous expression. The catalytic subunit of a bacterial GDH from *Burkholderia cepacia *was successfully expressed in *E. coli. *[11]. In contrast, expression levels and productivity for five putative FAD-dependent GDHs from several *Aspergillus species *in *E. coli *varied significantly [12]. To our knowledge no eukaryotic expression system was tested and published so far for the expression of FAD-dependent GDHs. We demonstrate that *G. cingulata *GDH (*Gc*GDH) can be heterologously expressed in *P. pastoris *as well as in *E. coli*, but with a big difference in the efficiency - expression levels are much higher for the eukaryotic system. In addition, recombinant GDH was compared with the enzyme isolated from its natural source to investigate if their differences in molecular and catalytic properties.

## Results

### Expression of *G. cingulata *glucose dehydrogenase in *E. coli*

To evaluate the influence of the N-terminal *Gc*GDH sequence on the amount of soluble, active *Gc*GDH expressed in *E. coli*, three nucleotide sequences coding for GDH with varying N-termini were cloned into pET-21a(+) for expression in *E.coli *under control of the T7 promoter. Plasmid GC1 encodes the full length *Gc*GDH including its native signal sequence. For plasmid GC2 the nucleotide sequence of the mature protein was cloned right after the start codon, and GC3 contains a truncated version starting 8 amino acids upstream of the FAD binding motif (GXGXXG). The resulting expression vectors were transformed into *E. coli *expression strains Rosetta 2, T7 Express and T7 Express (pGro7), and cells carrying the plasmids were cultivated in MagicMedia *sic! *at 20°C. Cultures were harvested at an optical density at 600 nm of approximately 15 and disrupted using a French press. The protein concentration of the cleared lysate varied between 6 to 12 mg mL^-1^. Lysates were tested for GDH activity using the standard DCIP enzyme assay.

Under the tested conditions active *Gc*GDH could only be detected in the T7 expression strains co-transformed with the plasmid pGro7 coding for chaperones. Of the three tested constructs, GC1 showed the highest volumetric activity (10 U L^-1 ^(DCIP); 5.5 U L^-1 ^(FcPF_6_)) in the fermentation medium supplemented with L-arabinose. GDH activity was lower (3.3 DCIP U L^-1^; 2.0 FcPF_6 _U L^-1^) for GC2 and no detectable GDH activity was measured for GC3. Activities were around five times lower without arabinose induction of the chaperones. The cell pellet obtained after disruption was tested for the existence of inclusion bodies using SDS-PAGE. The majority of proteins found in the insoluble fraction were of the molecular mass of *Gc*GDH (68 kDa). Refolding experiments were performed with inclusion bodies obtained from the expression experiment yielding the highest amount of soluble GDH. Samples were taken after 1, 12, 24 and 48 h of incubation in various refolding solutions containing FAD, but no activity could be detected from the tested refolding conditions.

### Production and purification of recombinant *Gc*GDH in *P. pastoris*

The *P. pastoris *expression plasmid pPIC*Gc*GDH was constructed by cloning the nucleotide sequence including the native *Gc*GDH signal sequence into the pPICZαA expression vector under control of the methanol-inducible AOX promoter. Transformed *P. pastoris *X-33 cells were checked for integration of the expression cassette into the genome by colony-PCR, and five positive transformants were tested for expression in a small-scale experiment. The best producing clone pPIC-GC1 (2400 U L^-1 ^GDH activity) was selected for further studies.

**Figure 1:**
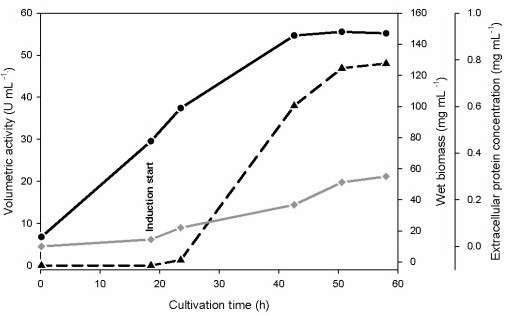
**Production of recombinant *Glomerella cingulata *GDH in *P. pastoris***. The yeast was cultivated in a 7-L bioreactor. The induction was started by a methanol feed phase. Black circles, wet biomass; black triangles, volumetric activity; grey diamonds, extracellular protein concentration.

Production of the enzyme was carried out in a 7-liter stirred and aerated bioreactor (Figure 1). The initial glycerol batch phase lasted for 19.5 h and produced 66.6 g L^-1 ^of wet biomass. During the 4 hours of the transition phase from glycerol to methanol the wet biomass further increased up to 99 g L^-1^. At this time a volumetric activity of 1900 U L^-1 ^was already detected. After the transition phase, a methanol feed was started and regulated manually to maintain a steady DO reading of 15%. Levels of wet biomass reached 149 g L^-1 ^during this induction phase, and the concentration of soluble protein in the culture supernatant increased from 80 to 300 mg L^-1^. Volumetric GDH activity in the culture supernatant reached a maximum value of 48,000 U L^-1^, corresponding to 57 mg of *recGc*GDH per litre of medium. After 50.5 h the fermentation was ended since the specific GDH activity in the culture supernatant started to decline.

The recombinant enzyme was purified to homogeneity using a two-step purification protocol employing hydrophobic interaction chromatography and anion exchange chromatography (Table 1). Strict pooling of only the purest fractions resulted in a moderately high yield of 71%. After purification, a bright-yellow protein solution was obtained and the purity was analyzed by SDS-PAGE. The final recombinant GDH preparation had a specific activity of 836 U mg^-1^.

**Table 1:** Purification of recombinant *Glomerella cingulata *glucose dehydrogenase.

Purification step	Total activity (U)	Total protein (mg)	Specific activity (U mg^-1^)	Yield (%)	Purification (fold)
Clear supernatant	215,000	1,300	165	100	1
Phenyl-Sepharose	160,000	192	833	74	5
DEAE-Sepharose	152,000	182	836	71	5.1

### Molecular and catalytic properties

**Figure 2:**
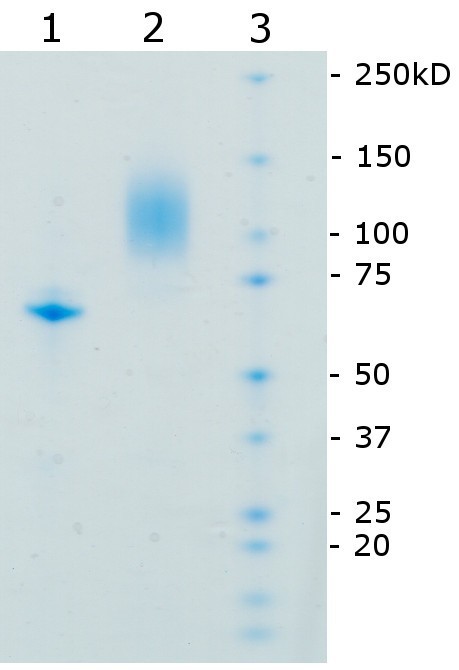
**SDS-PAGE analysis of glycosylated and deglycosylated recombinant GDH expressed in *P. pastoris***. *Lane 1, deglycosylated rec*Gc*GDH; lane 2, rec*Gc*GDH; lane 3, molecular mass marker*.

**Figure 3:**
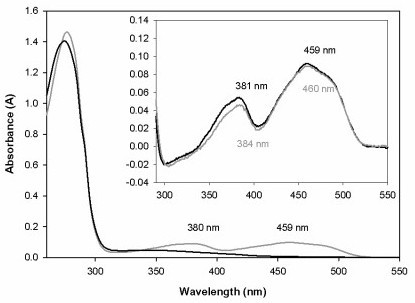
**Spectral characterization of GDH showing both the oxidized (gray) and reduced (black) spectra**. Glucose was used to reduce the enzyme. The difference spectra (ox-red) of *rec*GDH (black) and *wt*GDH (gray) are given as inset.

The molecular mass of *recGc*GDH produced in *P. pastoris *was determined by SDS-PAGE, which showed a broad and diffuse band between 88 and 131 kDa (Figure 2). After deglycosylation under denaturing conditions using PNGase F, a single, sharp band with an estimated molecular mass of 67 kDa was obtained. The typical flavoprotein spectrum shows the same characteristics as the spectrum of wild-type *Gc*GDH with almost identical FAD absorption maxima at 381 and 459 nm (Figure 3). These peaks disappear upon reduction of the enzyme by adding D-glucose.

The thermal stability of the *recGc*GDH was preliminary investigated by determining the temperature optimum which was found at 46°C. For GDH expressed by *G. cingulata *(produced according to [10]) the temperature optimum was 48°C. In a more detailed investigation using the ThermoFAD technique to derive thermal unfolding transition values (Tm) for different pH values and buffer substances (Table 2), *recGc*GDH showed a pH-dependent thermal stability with the highest Tm values in the acidic range of 4.5 to 6.4. The maximum Tm value of 56°C was measured in 50 mM sodium acetate buffer pH 5.0 and in 50 mM MES buffer pH 5.8. The activation energy was calculated to be 19.5 kJ mol^-1 ^from initial rates in the range of 26 to 51°C and is quite similar to the naturally produced GDH (21 kJ mol^-1^).

**Table 2:** Buffers and pH values used for the analysis of thermal stability (Tm) of *G. cingulata *GDH using *Thermo*FAD analysis [23].

Buffer	pH	Tm (°C)	pH	Tm (°C)	pH	Tm (°C)
Sodium acetate	**4.5**	55.0	**5.0**	56.0		
Sodium citrate	**4.7**	55.0	**5.5**	54.0		
Potassium phosphate	**5.0**	55.5	**6.0**	53.5	**7.0**	47.5
Sodium phosphate	**5.5**	54.5	**6.5**	51.0	**7.5**	45.0
MES	**5.8**	56.0	**6.2**	54.0	**6.5**	53.0
HEPES	**7.0**	50.5	**8.0**	43.0		
Ammonium acetate	**7.3**	52.5				
TRIS - HCl	**7.5**	47.0	**8.0**	42.5	**8.5**	38.5
Imidazole - HCl	**8.0**	41.5				
Bicine - HCl	**8.0**	44.0	**9.0**	36.0		

The buffers were each 50 mM.

The kinetic properties of *recGc*GDH were determined for the two best substrates that were identified for wild-type GDH, D-glucose and D-xylose. In these experiments ferrocenium was used as electron acceptor in saturating concentrations. The apparent catalytic constants were determined both at pH 5.5 and 7.5 and compared with those measured for GDH isolated from its natural source *G. cingulata *(Table 3, [10]). The molecular and catalytic properties of the recombinant enzyme overexpressed in *P. pastoris *are identical to those of the wild-type enzyme.

**Table 3:** Apparent kinetic constants of recombinant and wild-type *Glomerella cingulata *GDH for either D-glucose or D-xylose as substrate, with the concentration of the electron acceptor ferrocenium ion held constant at 20 μM.

Substrate and pH	enzyme	K_m_ (mM)	k_cat_ (s^-1^)	k_cat_/K_m_ (M^-1 ^s^-1^)
Glucose, pH = 5.5	wt	10.2 ± 0.2	180 ± 3	17.6 × 10^3^
	rec	10.1 ± 0.4	179 ± 4	17.7 × 10^3^

Glucose, pH = 7.5	wt	19.0 ± 0.3	380 ± 6	20.0 × 10^3^
	rec	17.1 ± 0.7	418 ± 4	24.5 × 10^3^

Xylose, pH = 5.5	wt	21 ± 0.6	40 ± 1.5	1.90 × 10^3^
	rec	26 ± 2.7	53 ± 1.9	2 × 10^3^

Xylose, pH = 7.5	wt	24 ± 1.5	60 ± 2	2.5 × 10^3^
	rec	23 ± 0.7	61 ± 1	2.7 × 10^3^

Kinetic data were determined at 30°C, the data for wild-type *Gc*GDH are from [10].

## Discussion

Recently the purification and characterization of a novel FAD-dependent glucose dehydrogenase produced by the plant pathogenic fungus *G. cingulata *and its proposed role in plant pathogenicity were published [10]. The reported features of this GDH are of interest in two respects: (i) to elucidate the role in the mechanism of plant-pathogen interactions during the infection process and (ii) in electrochemical applications [13,14]. To facilitate biochemical and structural studies as well as engineering of *G. cingulata *FAD-dependent GDH, the heterologous expression of *Gc*GDH was investigated. To target potential problems with the expression of a heavily glycosylated eukaryotic flavoprotein in a prokaryotic host several approaches were taken. Along with expression of *Gc*GDH with varying N-termini under mild conditions (auto inducing minimal media, 20°C) we also tested different *E. coli *expression strains for their suitability to express soluble and catalytically active *Gc*GDH.

The effect of the N-terminal amino acids on the expression levels of a fungal FAD-dependent GDH in *E. coli *was shown in the US patent 7,741,100 [15]. Expression levels could be increased approximately 10-fold by deletion of the signal sequence of *A. oryzae GDH*. Therefore, *Gc*GDH was expressed in full length and with the native signal sequence removed. A third, truncated N-terminus was designed according to a sequence alignment of closely related members of the GMC oxidoreductase family. The N-terminal sequences that were successfully used for the expression of *A. oryzae *GDH [15] and the flavin domain of *Phanerochaete chrysosporium *cellobiose dehydrogenase (CDH) in *E. coli *[16] seem to be highly conserved in these closely related proteins. The analogous sequence MTAYDYIVI was therefore chosen as N-terminal sequence for the third variant of *Gc*GDH. Surprisingly, although in a prokaryotic expression host, expression levels of *Gc*GDH were highest with the full-length protein, which included its own signal sequence. For the variant lacking the signal sequence the volumetric activity decreased three-fold, and no activity was detected for the third and shortest construct. For all tested expression constructs the fraction of GDH protein found in inclusion bodies (as judged by SDS-PAGE) was high. For the rather closely related *P. amagasakiense *glucose oxidase (GOX) refolding experiments from inclusion bodies were successful, retrieving ~10% of the totally aggregated GOX in an active form [17]. Although the same or slightly modified conditions were used, the same result could not be reproduced for *Gc*GDH. We conclude, that although GOx is the phylogenetically closest relative of GDH [10], the structure of GDH is different enough not to favour cofactor reconstitution under the same or similar conditions.

In addition to *in vitro *refolding of incorrectly folded protein several other methods have been described in literature for promoting the synthesis of active recombinant protein in the soluble cytoplasmic fraction rather than as inclusion bodies [18,19]. Increased amounts of the chaperone system GroEL/GroES in the cytoplasm apparently reduces the accumulation of aggregated *Gc*GDH in the cell, leading to small amounts of active soluble *Gc*GDH. The supply of tRNAs for 7 rare codons by the strain Rosetta 2, showed no beneficial effect on the expression of *Gc*GDH. This, however, was to be expected since codon analysis of the *gcgdh *gene revealed no sequences that could affect the transcriptional or translational efficiencies.

A further strategy to reduce the *in vivo *aggregation of recombinant *Gc*GDH in *E. coli *was to use slow growth and weak inducing conditions. To this end, the cultivation temperature was lowered to 20°C and an auto-inducing medium (MagicMedia) was used. It was shown previously that yields of a target protein as well as cell mass can be increased substantially by using such mild conditions [20]. Cell densities were increased up to 30 g L^-1 ^compared to 10 g L^-1 ^obtained by the standard LB medium. Even though all these considerations were taken into account for the expression of *Gc*GDH in *E. coli *a volumetric activity of10 U L^-1 ^could be produced under optimized conditions. Since expression rates in *P. pastoris *were much higher no effort was made to purify *Gc*GDH from *E. coli *cultures.

When using the eukaryotic expression system, *Gc*GDH could be expressed extracellularly in high yields using the native signal sequence, which indicates that this signal sequence is properly recognized and processed by the yeast. A final volumetric activity of 48,000 U L^-1 ^and a space-time yield of 24 mg L^-1 ^d^-1 ^could be achieved by *P. pastoris*. This is a 70-fold improvement of the space-time yield compared to the wild-type producer. The cultivation yielded a total of 57 mg L^-1 ^of recombinant protein, which corresponds to ~20% of total extracellular protein. The purification protocol resulted in a protein preparation of high purity (as checked by SDS-PAGE) with a specific activity of 836 U mg^-1^, which is comparable to the wild type preparation (840 U mg^-1^,^,^[10]). Since the first purification step already yielded a protein of high specific activity (833 U mg^-1^) the procedure might be reduced to a one-step purification. All (bio)physical and catalytic properties studied for *recGc*GDH are essentially identical to those of the wild-type enzyme isolated from the original source *G. cingulata *(Table 3, [10]**)**. The high degree of glycosylation of recombinant GcGDH (approx. 65% as judged from SDS-PAGE, Figure 2) is also found in *native Gc*GDH (approx. 70%, [10]). These values are certainly an overestimation by SDS-PAGE, which is known to smear bands of glycosylated proteins, but the range of the bands of native (95-135 kDa) and recombinant (88-131 kDa) GcGDH are nearly identical. The temperature optimum for *recGc*GDH is 46°C and close to the previously reported value for an FAD-dependent glucose dehydrogenase from *A. terreus *(50°C) [14].

This study reports and compares the successful heterologous expression of *Glomerella cingulata *GDH in *P. pastoris *and *E. coli*. The glycosylation of this protein seems to play an important role for folding into the correct conformation, as already shown for other proteins as well [21]. This makes the eukaryotic host more suitable for the production of *recGc*GDH, which displays properties that are essentially identical to those of the wild-type enzyme [10]. The expression in *E. coli *has the advantage that glycosylation-free *Gc*GDH can be obtained, which is useful for e.g. crystallization studies. However, for this application the production in the prokaryotic host has to be optimized further to provide sufficient amounts of protein.

## Conclusions

The suitability of a eukaryotic and a prokaryotic expression system for the heterologous overexpression of an extracellular fungal glucose dehydrogenase is tested by this study. The expression of *Gc*GDH in *P. pastoris *provides a suitable method for the easy preparation of sufficient amounts of GDH as well as genetically engineered GDH variants for further applications in electrochemistry, for structure/function studies or for the study of plant-pathogen interactions of this attractive novel enzyme.

## Methods

### Strains and media

*P. pastoris *X-33 is a component of the EasySelect *Pichia *Expression Kit and was obtained from Invitrogen. Chemical competent *E. coli *strain NEB 5-alpha was purchased from New England Biolabs (NEB) and used for maintenance and propagation of plasmids. *E. coli *expression strains Rosetta 2 and T7 Express were ordered from Novagen and from New England Biolabs, respectively. *E. coli *cells were cultivated in LB-medium (peptone from casein 10 g L^-1^, yeast extract 5 g L^-1^, NaCl 10 g L^-1^) containing 100 mg L^-1 ^ampicillin and/or 30 mg L^-1 ^chloramphenicol. Low Salt LB-medium (NaCl reduced to 5 g L^-1^) was used when zeocin (25 mg L^-1^) was used as selection marker. MagicMedia *sic! E. coli *expression medium (Invitrogen) was used for expression studies in *E. coli. P. pastoris *transformants were grown on YPD plates (yeast extract 10 g L^-1^, peptone 20 g L^-1^, dextrose 10 g L^-1^, zeocin 100 mg L^-1^) and the Basal Salts Medium (Invitrogen) was used for fermentation.

### Chemicals and Vectors

All chemicals were purchased from Sigma, Fluka, Roth or VWR and were of the highest purity available. Primers were from VBC-Biotech and nucleotide sequences are shown in Table 4. Restriction enzymes and T4-ligase were purchased from Fermentas, Phusion polymerase from NEB and the yeast expression vector pPICZαA from Invitrogen. The plasmid pET-21a(+)from Novagen was used for expression in *E. coli*. Plasmid pGro7 encoding the chaperones GroEL and GroES was purchased from TAKARA Bio Inc. (Japan).

**Table 4:** Nucleotide sequences of primers.

Primer name	Sequence (from 5' to 3')
GC-GDH*nde*Ifw1	TATCATATGAAGAACCTCATTCCTC
GC-GDH*nde*Ifw2	TATCATATGCCAGGTTCTGCCCCCAGGG
GC-GDH*nde*Ifw3	TATCATATGACGGCATACGACTATATTGTC
GC-GDH*not*Irv	ATACGGCCGTCATTAAGCAGCAGCCTTGATCAGAT
GC-GDH-*Bst*BI+SS	TATTTCGAAATGAAGAACCTCATTCCTCTTTCC
GC-seq-rv1	AGGTAGAAGCACCACCAGAGG

### Heterologous expression in *E. coli*

The published plasmid pGC1 [10] was used as template for the amplification of *Gc*GDH cDNA (JF731352) with three different forward primers (GC-GDH*nde*Ifw1 - 3) and the reverse primers GC-GDH*not*Irv1. The three resulting nucleotide sequences encoded *Gc*GDH with varying N-termini. Both the PCR fragments and the expression vector pET-21a(+) were digested with *Nde*I and *Not*I and ligated using the Rapid DNA Ligation Kit from Fermentas. Correct insertion of the genes and the absence of mutations were checked by DNA sequencing and verified plasmids were transformed into *E. coli *Rosetta 2, *E. coli *T7 Express and *E. coli *T7 Express carrying the plasmid pGro7. In order to compare the expression levels of *Gc*GDH with these 9 different expression strategies, small-scale cultivation in 125-mL baffled shaken flasks filled with 30 mL media were performed at 20°C. To reduce time-consuming steps such as monitoring optical density (OD) prior to induction or adding appropriate inducers, the autoinducing MagicMedia (Invitrogen) was used for this comparative study. Chaperone co-expression was tested both with 1 mg mL^-1 ^L-arabinose for induction and without added inducer.

All cultures were grown at 37°C for 5 h and then further cultivated overnight at 20°C. Cell suspensions were centrifuged at 4000 × *g *for 10 min at 4°C, the cell pellets were suspended in lysis buffer (50 mM potassium phosphate buffer pH 6.5 supplemented with 5.7 mM PMSF), and disrupted by using a French Press. The crude extract was cleared by centrifugation (4000 × *g*, 30 min, 4°C), the supernatant was tested for GDH activity by the colorimetric DCIP assay, and the pellet was analyzed for insoluble GDH by SDS-PAGE. Refolding experiments were done according to the protocol of the Renaturation Basic Kit for Proteins (Sigma). Additionally, flavin adenine dinucletide (FAD) was added to the renaturing solution at a concentration of 50 μM.

### Heterologous expression in *Pichia pastoris*

*Gc*GDH-encoding cDNA was amplified using the primers GC-GDH-*BstB*I+SS and GC-GDH-*Not*I. The PCR amplicon was digested with *Bsp119*I and *Not*I and cloned into the yeast expression vector pPICZαA. The resulting plasmid pPIC*Gc*GDH was linearized with *Mss*I and transformed into electrocompetent *P. pastoris *X-33 cells prepared according to the operating instructions and applications guide of the MicroPulser electroporation apparatus (Biorad). Transformants were selected on YPD zeocin plates, and the integration of the gene was checked by colony PCR with the primers AOX-fw and GC-seq-rv1. Five positive colonies were selected for expression studies in baffled shaken flasks. Pre-cultures (50 mL) were grown overnight at 30°C in YPD medium containing 50 mg L^-1 ^zeocin. After approximately 16 h of growth the pre-cultures were transferred into 1-L baffled shaken flasks containing 300 mL of BMMY medium. Methanol (0.5% v/v final concentration) was added regularly (approximately every 12 h) while incubating at 30°C and shaking at 150 rpm. Samples were taken every day and analyzed for protein concentration and GDH activity.

### Enzyme production and purification

Recombinant *Gc*GDH was produced in a 7-L glass vessel fermenter (MBR) filled with 4 L of medium (Basal Salts Medium). After autoclaving, the pH of the medium was adjusted to 5.0 with 28% ammonium hydroxide and maintained at this pH for the entire fermentation process. The fermentation was started by adding 0.4 L (9% v/v) of preculture grown on YPD medium in 1-L baffled shaken flasks at 125 rpm and 30°C overnight. The cultivation was executed according to the *Pichia *Fermentation Guideline of Invitrogen and enzyme production was induced with methanol. At the transition phase from glycerol to methanol feed the protocol was altered according to Zhang *et al. *[22]. At the end of the glycerol batch phase methanol (0.2% v/v) was injected aseptically into the fermenter, and the glycerol feed faded out by a linear ramp 20 g L^-1 ^h^-1 ^to 0 g L^-1 ^h^-1 ^over 4 h. Once the dissolved oxygen concentration spiked, the methanol feed was started. It was regulated to keep a stable dissolved oxygen concentration of 15%. The cultivation temperature was 30°C, the variable airflow rate was around 6 L min^-1^, and the agitation was set to 800 rpm. Samples were taken regularly and clarified by centrifugation. The pellet was used to determine wet biomass. GDH activity and extracellular protein concentration were assayed in the supernatant.

The fermentation broth was clarified by centrifugation (6000 × g; 30 min; 4°C) and saturated ammonium sulfate solution was slowly added to give a 60% saturated solution. Precipitates were removed by ultracentrifugation (30,000 × g; 15 min; 4°C) and the enzyme was purified by hydrophobic interaction chromatography on a 400-mL PHE Sepharose 6 fast flow column (chromatographic equipment and materials from GE Healthcare Biosciences) equilibrated with 50 mM phosphate buffer pH 7 containing 60% (saturation) ammonium sulfate. Proteins were eluted within a linear gradient from 60 to 0% ammonium sulfate in 8.5 column volumes (CV, 3.4 L) and collected in 50 mL fractions. Active fractions were pooled and diafiltrated using a hollow fiber cross-flow module (Microza UF module SLP-1053, 10 kDa cut-off, Pall Corporation). The partially deionized enzyme solution (3 mS cm^-1^) was applied to a column packed with 100 mL DEAE-Sepharose FF, previously equilibrated with 50 mM phosphate buffer, pH 7.5. Proteins were eluted within a linear salt gradient from 0 to 2 M NaCl in 10 CV (1 L). The pooled fractions were concentrated and the buffer was exchanged by diafiltration to 50 mM MES pH 5.8, and the enzyme solution was filter sterilized, aliquoted and stored at -30°C.

### Enzyme assays and protein determination

Glucose dehydrogenase activity was assayed spectrophotometrically using 2,6-dichloroindophenol (DCIP, ε_520 _= 6.9 mM^-1 ^cm^-1^) as electron acceptor. The reaction was followed for 180 s at 30°C in a Lambda 35 UV/Vis spectrophotometer (Perkin Elmer). The DCIP-based assay contained (final concentrations) 50 mM sodium acetate buffer, pH 5.5, 300 μM DCIP and 100 mM D-glucose. Alternatively, ferrocenium hexafluorophosphate (ε_300 _= 4.3 mM^-1 ^cm^-1^) was used as electron acceptor for the determination of the catalytic constants to enable measurements in the range of pH 5.5 and 7.5. One unit of GDH activity was defined as the amount of enzyme necessary for the reduction of 1 μmol glucose or electron acceptor per min under the assay conditions [10]. It is noted that DCIP is a two-electron acceptor, but the ferrocenium ion a one-electron acceptor. The protein concentration was determined by the method of Bradford using a prefabricated assay (Bio-Rad) and bovine serum albumin as standard.

### Molecular properties

SDS-PAGE was carried out using Mini-PROTEAN TGX precast gels with a denaturing gradient of 4-15%. Protein bands were visualized by staining with Bio-Safe Coomassie (Bio-Rad). Dual Color Precision Plus Protein Standard (Bio-Rad) was used for mass determination. All procedures were done according to the manufacturer's recommendations. To estimate the degree of glycosylation homogenous *recGc*GDH was treated with PNGase F (NEB) under denaturing conditions according to the manufacturer's instructions. The spectrum of homogeneously purified *recGc*GDH was recorded at room temperature from 250 to 550 nm in both the oxidized and reduced state using a U-3000 Hitachi spectrometer (Tokyo, Japan). GDH was diluted in 50 mM citrate buffer, pH 5.5 to an absorbance of ~1.5 at 280 nm and the spectrum was recorded before and shortly after the addition of glucose to the cuvette. The temperature profile of activity for wildtype and recombinant GDH was determined in parallel by measuring the average GDH activity over 5 min from 25 to 62°C in temperature controlled DCIP assays.

### *Thermo*FAD analysis

The Thermofluor-based *Thermo*FAD method [23] was used to monitor protein unfolding for analysis of thermal stability of *recGc*GDH in a set of 22 different buffers, each at 50 mM, over a pH range from pH 4.5-9.0. Buffers used can be seen in Table 4. The method takes advantage of the intrinsic fluorescence of the FAD cofactor, and does not depend on fluorescent dyes. *recGc*GDH was diluted in buffer to a final concentration of 1 mg mL^-1 ^and subsequently analyzed in triplicates in 50 μL aliquots per well. A real-time PCR cycler (i-Cycler, Bio-Rad) providing a MyiQ Optics Module, and SYBR-Green filters (523-543 nm) was used to record the signals. The samples were heated in 0.5°C steps (20 s per step) from 30° to 95°C. The fluorescence signal was measured at the end of each step.

### Steady-state kinetics

Apparent kinetic constants for D-glucose and D-xylose were determined with ferrocenium hexafluorophosphate as electron acceptor at a fixed concentration of 200 μM using glucose in the range of 1-100 mM, and xylose in the range of 100-1500 mM. Constants were calculated using nonlinear least-squares regression by fitting the observed data to the Michaelis-Menten equation (Sigma Plot 11, Systat Software).

## Competing interests

The authors declare that they have no competing interests.

## Authors' contributions

CS and RL drafted the outline of the expression experiments, protein purification and characterization. PS and NP carried out the construction of the expression vectors and PS performed *E. coli *expression studies. MK conducted the *P. pastoris *fermentation, GDH purification and characterization. NP and KDj-C helped with the selection of expression vectors, strains and cultivation conditions and participated in stability studies. LG suggested stability experiments and interpreted the data. CS wrote the first draft of the manuscript. KDj-C and LG revised the manuscript. RL and DH coordinated the study, verified and interpreted results and revised the final manuscript. All authors have read and approved the final manuscript.

## References

[B1] Ogura Y Studies on the glucose dehydrogenase of *Aspergillus oryzae* J Biochem 1951 38 1 75 84

[B2] Sode K Tsugawa W Yamazaki T Watanabe M Ogasawara N Tanaka M A novel thermostable glucose dehydrogenase varying temperature properties by altering its quaternary structures Enzyme Microb Technol 1996 19 2 82 85 10.1016/0141-0229(95)00170-0

[B3] Lovallo N Cox-Foster DL Alteration in FAD-glucose dehydrogenase activity and hemocyte behavior contribute to initial disruption of *Manduca sexta *immune response to *Cotesia congregata *parasitoids J Insect Physiol 1999 45 12 1037 1048 10.1016/S0022-1910(99)00086-4 12770264

[B4] Cavener DR MacIntyre RJ Biphasic expression and function of glucose dehydrogenase in *Drosophila melanogaster* Proc Natl Acad Sci USA 1983 80 20 I 6286 6288 6413974 10.1073/pnas.80.20.6286 PMC394281

[B5] Okuda-Shimazaki J Kakehi N Yamazaki T Tomiyama M Sode K Biofuel cell system employing thermostable glucose dehydrogenase Biotechnol Lett 2008 30 10 1753 1758 10.1007/s10529-008-9749-7 18516502

[B6] Omura H Sanada H Yada T Morita T Kuyama M Ikeda T Kano K Tsujimura S Coenzyme-binding glucose dehydrogenase 2004 In EP 1 584 675

[B7] Bak T-G Sato R Studies on the glucose dehydrogenase of *Aspergillus oryzae*: I. Induction of its synthesis by p-benzoquinone and hydroquinone Biochim Biophys Acta, Enzymol 1967 139 2 265 276 10.1016/0005-2744(67)90031-9 4962256

[B8] Tsuji Y Kitabayashi M Kishimoto T Nishiya Y Glucose dehydrogenase from *Aspergillus oryzae* 2010 In US patent 7,655,130 B2

[B9] Aiba H Tsugura-shi J Novel glucose dehydrogenase US Patent 2007/0105174

[B10] Sygmund C Klausberger M Felice A Ludwig R Reduction of quinones and phenoxy radicals by extracellular glucose dehydrogenase from *Glomerella cingulata *suggests a role in plant pathogenicity Microbiology 2011 157 11 3203 3212 10.1099/mic.0.051904-0 21903757

[B11] Inose K Fujikawa M Yamazaki T Kojima K Sode K Cloning and expression of the gene encoding catalytic subunit of thermostable glucose dehydrogenase from *Burkholderia cepacia *in *Escherichia coli* Biochim Biophys Acta - Proteins & Proteomics 2003 1645 2 133 138 10.1016/S1570-9639(02)00534-4 12573242

[B12] Mori K Nakajima M Kojima K Murakami K Ferri S Sode K Screening of *Aspergillus*-derived FAD-glucose dehydrogenases from fungal genome database Biotechnology Letters 1 9 10.1007/s10529-011-0694-5 21748361

[B13] Heller A Feldman B Electrochemical glucose sensors and their applications in diabetes management Chemical Reviews 2008 108 7 2482 2505 10.1021/cr068069y 18465900

[B14] Tsujimura S Kojima S Kano K Ikeda T Sato M Sanada H Omura H Novel FAD-Dependent Glucose Dehydrogenase for a Dioxygen-Insensitive Glucose Biosensor Biosci, Biotechnol, Biochem 2006 70 3 654 659 10.1271/bbb.70.654 16556981

[B15] Kitabayashi M Tsuji Y Kawarabayshi Y Kishimoto T Nishiya Y Method for highly expressing recombinant glucose dehydrogenase derived from filamentous fungi 2010

[B16] Desriani Ferri S Sode K Functional expression of *Phanerochaete chrysosporium *cellobiose dehydrogenase flavin domain in *Escherichia coli* Biotechnology Letters 32 6 855 859 10.1007/s10529-010-0215-y 20140751

[B17] Witt S Singh M Kalisz HM Structural and kinetic properties of nonglycosylated recombinant *Penicillium amagasakiense *glucose oxidase expressed in *Escherichia coli* Applied and Environmental Microbiology 1998 64 4 1405 1411 9546178 10.1128/aem.64.4.1405-1411.1998 PMC106162

[B18] Sørensen H Mortensen K Soluble expression of recombinant proteins in the cytoplasm of *Escherichia coli* 2005 4 1 10.1186/1475-2859-4-1 PMC544838 15629064

[B19] Sørensen HP Mortensen KK Advanced genetic strategies for recombinant protein expression in *Escherichia coli* Journal of Biotechnology 2005 115 2 113 128 10.1016/j.jbiotec.2004.08.004 15607230

[B20] Studier FW Protein production by auto-induction in high density shaking cultures Protein expression and purification 2005 41 1 207 234 10.1016/j.pep.2005.01.016 15915565

[B21] Shental-Bechor D Levy Y Effect of glycosylation on protein folding: A close look at thermodynamic stabilization Proceedings of the National Academy of Sciences of the United States of America 2008 105 24 8256 8261 10.1073/pnas.0801340105 18550810 PMC2448824

[B22] Zhang W Inan M Meagher MM Fermentation strategies for recombinant protein expression in the methylotrophic yeast *Pichia pastoris* Biotechnology and Bioprocess Engineering 2000 5 4 275 287 10.1007/BF02942184

[B23] Forneris F Orru R Bonivento D Chiarelli LR Mattevi A ThermoFAD, a Thermofluor^®^-adapted flavin ad hoc detection system for protein folding and ligand binding FEBS Journal 2009 276 10 2833 2840 10.1111/j.1742-4658.2009.07006.x 19459938

